# Brain-expressed 3′UTR extensions strengthen miRNA cross-talk between ion channel/transporter encoding mRNAs

**DOI:** 10.3389/fgene.2014.00041

**Published:** 2014-02-26

**Authors:** Claudia C. Wehrspaun, Chris P. Ponting, Ana C. Marques

**Affiliations:** ^1^Department of Physiology, Anatomy and Genetics, University of OxfordOxford, UK; ^2^Section on Neuropathology, Clinical Brain Disorders Branch, Genes, Cognition and Psychosis Program, IRP, NIMH, National Institutes of HealthBethesda, MD, USA; ^3^MRC Functional Genomics Unit, Department of Physiology, Anatomy and Genetics, University of OxfordOxford, UK

**Keywords:** 3′untranslated region, 3′ UTR, gene expression, brain, miRNA, ion channels, transporters, competitive endogenous RNAs

## Abstract

Why protein-coding genes express transcripts with longer 3′untranslated regions (3′UTRs) in the brain rather than in other tissues remains poorly understood. Given the established role of 3′UTRs in post-transcriptional regulation of transcript abundance and their recently highlighted contributions to miRNA-mediated cross-talk between mRNAs, we hypothesized that 3′UTR lengthening enhances coordinated expression between functionally-related genes in the brain. To test this hypothesis, we annotated 3′UTRs of human brain-expressed genes and found that transcripts encoding ion channels or transporters are specifically enriched among those genes expressing their longest 3′UTR extension in this tissue. These 3′UTR extensions have high density of response elements predicted for those miRNAs that are specifically expressed in the human frontal cortex (FC). Importantly, these miRNA response elements are more frequently shared among ion channel/transporter-encoding mRNAs than expected by chance. This indicates that miRNA-mediated cross-talk accounts, at least in part, for the observed coordinated expression of ion channel/transporter genes in the adult human brain. We conclude that extension of these genes' 3′UTRs enhances the miRNA-mediated cross-talk among their transcripts which post-transcriptionally regulates their mRNAs' relative levels.

## Introduction

Post-transcriptional regulation of transcript abundance can be achieved by altering RNA stability or translation (Shyu et al., 2008). Untranslated regions (UTRs) located at 5′ and 3′ ends of mRNAs contain the majority of post-transcriptional regulatory elements. Of these, the 3'UTR contain the highest density of motifs contributing to mRNA stability, translation and localization (Kuersten and Goodwin, 2003; Wang et al., 2008). A pre-eminent class of such motifs are miRNA recognition elements (MREs) that bind to near complementary miRNAs (~22 nucleotide non-coding RNAs), which in turn induce either degradation or translational inhibition of their cognate mRNAs targets (Stark et al., 2005). Conserved miRNA sites in 3′UTRs experience stronger negative selection compared to other functional elements (Chen and Rajewsky, 2006). The significance of miRNAs in regulating transcript abundance is exemplified by the prediction of their MREs in over 60% of human genes (Bartel, 2009) and their demonstrated roles in many aspects of neuronal cell physiology, including terminal neuronal differentiation (Kawase-Koga et al., 2009), polarization of neurons (Wang et al., 2008), axonogenesis (Wu et al., 2012), neuroplasticity and stress response (Nelson et al., 2008).

Spatially and temporally regulated expression of alternative 3′UTRs, resulting either from alternative cleavage and polyadenylation or alternative splicing, are a common feature of animal genes (Iseli et al., 2002; Hughes, 2006). Alternative 3′UTR usage is often preserved across evolution as indicated by, for example, similar levels of between-species sequence conservation at proximal and distal poly(A) sites (Smibert et al., 2012). Differences in 3'UTR length are in many cases correlated with transcript abundance (Ulitsky et al., 2012). The 3′UTRs of transcripts that are expressed in the brain tend to be longer than those expressed in other tissues, a property that is widely conserved across evolution (Zhang et al., 2005; Ji et al., 2009; Hilgers et al., 2011; Ulitsky et al., 2012; Miura et al., 2013). For example transcripts whose proteins have roles in development, morphogenesis and signal transduction express longer 3′UTRs compared to transcripts for proteins involved in metabolism or RNA processing (Ramskold et al., 2009) and 3′UTRs of genes encoding ribosomal genes have been reported to be ~6-fold shorter than 3′UTRs of neurogenesis genes (Stark et al., 2005).

Here we sought to address why such transcripts might require longer 3′UTRs using an assumption that their greater lengths reflect a greater degree of post-transcriptional regulation by miRNAs. Long 3′UTRs of neuronally expressed isoforms are enriched in conserved binding sites (MREs) for well-known neural miRNAs (Miura et al., 2013) that are thought to contribute to the fine-tuning of their encoded products' abundance. For example, the predominant *MeCP2* isoform in human fetal brain contains an unusually long 3′UTR (Coy et al., 1999; Balmer et al., 2003) which harbors functional binding sites for miR-483-5p, a fetal brain-enriched miRNA that post-transcriptionally represses *MeCP2* in this tissue (Han et al., 2013). Interestingly, miR-483-5p also regulates transcript levels of other *MeCP2* protein interacting partners (Han et al., 2013). This suggests that differential 3′UTR usage may fine-tune gene-gene interactions via miRNA-mediated modulation of their transcripts' abundance (Han et al., 2013).

The ability of mRNAs to compete for miRNAs depends both on their relative abundance and their MRE repertoire (Ala et al., 2013). The latter is determined, largely, by the sequence and length of their 3′UTRs (Mangone et al., 2010) which control, in a miRNA-dependent manner, their relative abundance (Chi et al., 2009). We hypothesized that genes with brain-specific 3′UTR extensions might engage in the miRNA-mediated cross-talk with other functionally related transcripts. Given that there is a known excess of long alternative 3′UTRs in brain-expressed isoforms (Zhang et al., 2005; Ji et al., 2009; Hilgers et al., 2011; Ulitsky et al., 2012; Miura et al., 2013), we expected such transcripts to contribute to brain-specific biological processes.

A key feature of brain cells, particularly neurons, is their ability to respond to electrochemical changes in their extracellular environment (Holtmaat and Svoboda, 2009; Vaquerizas et al., 2009). Crucial to this process are genes encoding ion channels and transporters. These proteins form, often gated, multi-subunit pores in the membrane which permit efficient and specific transport of extracellular ions (Alberts et al., 2002). Ion channels and transporters are present in most cell types (Alberts et al., 2002) but their importance in the central nervous system is illustrated by the many channelopathies that affect these tissues (Jentsch et al., 2004). Importantly, the transcript abundance of ion channel encoding genes is influenced by environmental cues, including the electrical activity present within the cell (Schulz et al., 2008). Given that ion channels require the coordinated assembly of several protein subunits to convey their function, transcript abundance may be tightly regulated to ensure proper channel assembly and stoichiometry. Such coordination can be achieved via miRNA-mediated post-transcriptional regulation. For example, miR-103 simultaneously regulates the abundance of at least three transcripts encoding subunits of the Ca_v_1.2 L-type calcium channel, and modulates its activity (Favereaux et al., 2011). Variants in this channel play a role in chronic neuropathic pain (Favereaux et al., 2011) as well as in Timothy's (Splawski et al., 2006) and Brugada Syndrome (Antzelevitch et al., 2007).

Here we investigated the biological relevance of 3′UTR lengthening among transcripts expressed in the human adult brain. Genes that express their longest alternative 3′UTR in this organ were found to be substantially enriched for those encoding ion channels and transporters. We provide evidence that extended 3′UTR transcription in the brain provides an additional miRNA-mediated layer of post-transcriptional regulation of these genes' transcript levels.

## Materials and methods

### RNA sequencing data

We obtained single- and paired-end long poly-adenylated RNA sequencing (RNA-Seq) reads derived from 16 human adult tissues (one individual per tissue adipose, adrenal, blood, brain, breast, colon, heart, kidney, liver, lung, lymph-node, ovary, prostate, skeletal-muscle, testes, and thyroid) from the Illumina's Human BodyMap 2.0 project (ArrayExpress, 2011a). Details on the samples and methods employed in the preparation of this data can be found in (ArrayExpress, 2011b). The median coverage of constitutively expressed 3′UTR nucleotides was 13.5X.

The expression profiles of human miRNAs were obtained from (Landgraf et al., 2007) and used to annotate miRNA families expressed in human adult frontal cortex (FC). A miRNA was considered to be expressed, if it had ≥1 reads supporting its expression in this tissue (resulting in 76 expressed miRNA families Landgraf et al., 2007). A miRNA family was defined as FC-specifically expressed if its expression was supported by a number of reads which was 2-, 5-, or 10-times higher than this miRNA's median across all non-neuronal tissues (Supplementary Table S1) (Landgraf et al., 2007).

### Annotation of constitutive and alternative 3′untranslated regions

Single- and paired-reads for each of the 16 tissues were mapped to the human genome (hg19) using TopHat (Trapnell et al., 2009). Transcripts were assembled *de novo* with Cufflinks (version 1.3.0, Trapnell et al., 2010) using ENSEMBL (build 71, Flicek et al., 2012) protein-coding gene transcripts as reference. We considered transcripts that overlapped exonic bases of ENSEMBL protein-coding genes to be part of that gene locus.

**Figure 1:**
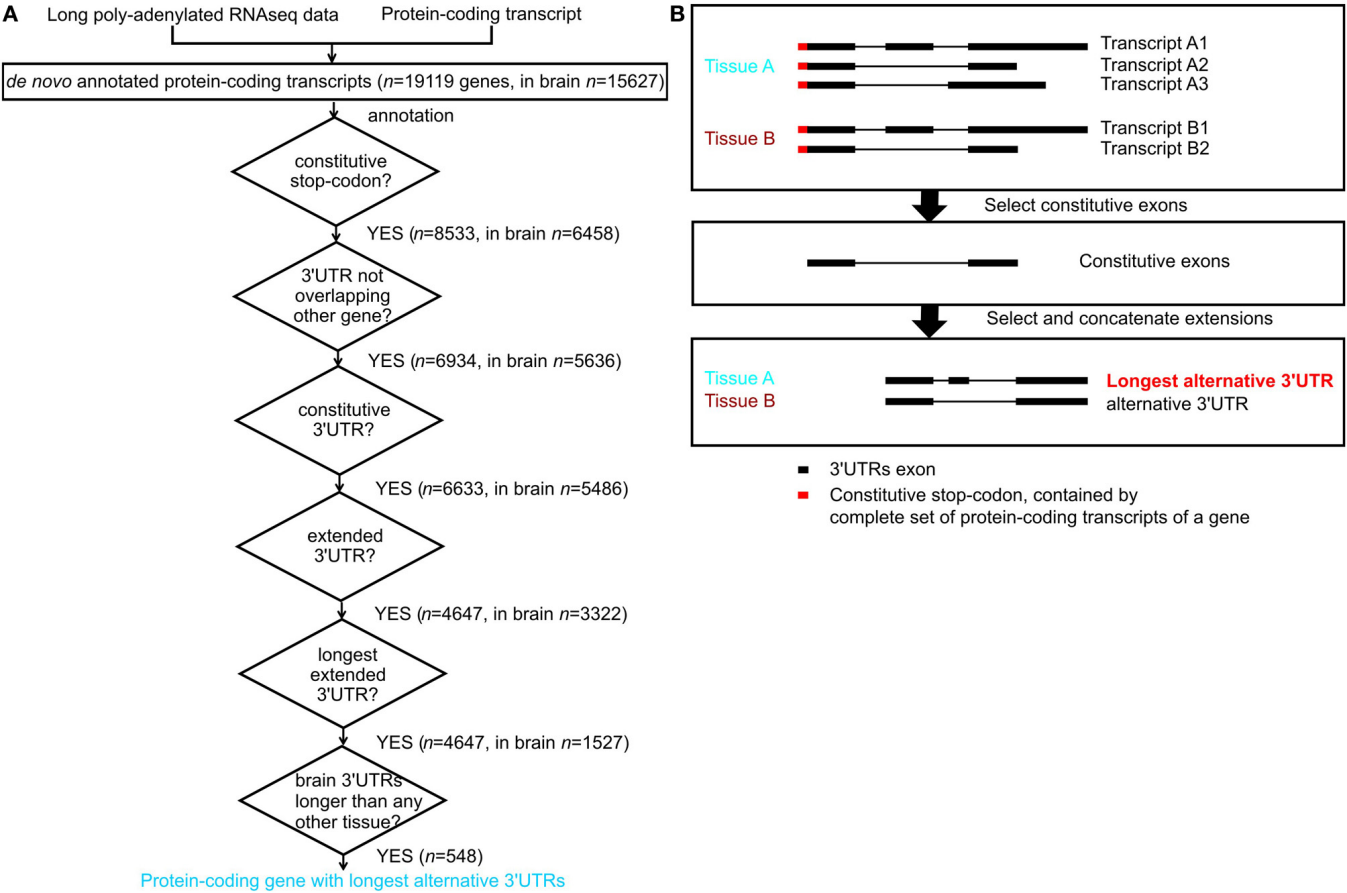
**Annotation of constitutive and alternative 3′UTRs. (A)** Flowchart for annotation of protein-coding genes with longest alternative 3′UTRs; *n* indicates number of genes. **(B)** Example of a gene with constitutive stop codon (red), expressed only in tissue A (blue) and tissue B (brown). Tissue A expresses three transcripts, and tissue B expresses 2 transcripts (top panel). The constitutive 3′UTRs consists in the nucleotides expressed in all transcript of a gene across all tissues where the gene is expressed (middle panel). The extended alternative 3′UTRs is the consensus transcript for a gene in a given tissue after constitutive parts have been removed. Tissue A expresses the longest alternative 3′UTRs for this gene, as it contains more nucleotides than the alternative 3′UTRs in tissue B (bottom panel).

For each tissue, we excluded protein-coding genes that were not expressed (have an FPKM -Fragments Per Kilobase of transcript per Million mapped reads- equal to zero). We included genes annotated in ENSEMBL and newly assembled transcripts. Next we considered only protein-coding genes with stop codon genomic locations that were common to all its annotated transcripts in ENSEMBL; we refer to these as constitutive stop codons (*n* = 8533 genes contained constitutive stop codons (Figure 1A) out of *n* = 19985 protein-coding genes with annotated stop codons in ENSEMBL build 71). For each gene and in each tissue, we annotated a consensus transcript, containing all transcribed nucleotides and defined its 3′UTR as the transcribed region downstream of its constitutive stop codon (Figure 1B). The nucleotide regions common to all transcripts from a gene were annotated as that gene's constitutive 3′UTRs and the remaining 3′UTR nucleotides were considered to be alternative 3′UTRs. For each gene, we annotated the alternative 3′UTRs with the maximal length in nucleotides as its longest alternative 3′UTRs (Figure 1B).

### 3′UTR expression

We used CoverageBed (Bedtools version 2.17.0; Quinlan and Hall, 2010) to determine the number of long mRNASeq reads derived from each adult human tissue mapping to constitutive and alternative 3′UTRs. For each tissue, we estimated constitutive and alternative 3′UTR expression (*E*) as the number of reads mapping to that region divided by its total length. To identify differences in 3′UTRs usage between tissues, we estimated the ratio between the expression in tissue *i* of the longest alternative (*L*) and constitutive (*C*) 3′*UTRs*(*E*_*i*_ = *E*_*Li*_/*E*_*Ci*_).

### Lineage specific constraint

We used the derived frequencies of 1000 Genomes single nucleotide polymorphisms (SNP; Clarke et al., 2012; Haerty and Ponting, 2013) to test for differences in selective constraint within the human lineage between ancestral repeats (AR), constitutive 3′UTRs and the longest alternative 3′UTRs across all tissues, as described in (Chen and Rajewsky, 2006; Marques and Ponting, 2009). We considered SNPs with frequency *f* < 10% to be rare and intermediate if 10% < *f* < 90%. We considered only ARs within the vicinity of 3′UTRs [20, 50, and 100 kilobases (kb) downstream of the alternative 3′UTRs or upstream of the constitutive stop codon].

### Functional enrichment

For each tissue, we tested whether genes with the longest alternative 3′UTR expressed in only that tissue were functionally enriched using the functional classification tool Database for Annotation, Visualization, and Integrated Discovery (DAVID; Huang et al., 2009) using default parameters (count = 2 and ease = 0.1). Genes expressed in only one tissue were discarded, for example in brain, out of *n* = 548 genes expressing their longest alternative 3′UTRs in brain only 4 were excluded. For each tissue, we compared the Gene Ontology (GO) annotations of genes with a longest alternative 3′UTR expressed in that tissue to a background containing all genes with annotated 3′UTRs expressed in that tissue. We considered GO-terms that were significantly enriched at α = 0.05, after Bonferroni multiple testing correction.

### Expression correlation

We tested whether the genes (*n* = 226) contributing to functional enrichment of ion channel and transporter encoding genes (referred to as “*ion channel/transporter genes*”) showed increased correlation in expression. In order to match the developmental stage of the subject from which the RNA used to annotate 3′UTRs in the brain was obtained (a 77 year old female) we restricted our analyses of gene expression to adulthood (20–78 years; *n* = 148) using the microarray data from “braincloud” (Colantuoni et al., 2011), preprocessed and quantified as described by the authors (Colantuoni et al., 2011). “Braincloud” contains gene expression data from the prefrontal cortex (PFC) of human postmortem tissue. For each gene, we used only one probe, namely the probe that showed the strongest correlation with age during the lifespan (Colantuoni et al., 2011) (number of probes = 16812) to avoid bias arising from using multiple probes for one gene. *N* = 192 probes were available for *ion channel/transporter genes*. We compared the median of all pairwise Spearman's correlations among the *ion channel/transporter genes* to the same measure from *n* = 1000 random bootstrap samples of same set size (*n* = 192 genes, matched to *ion channel/transporter genes*) from (i) brain expressed genes (*n* = 16812 probes available in “braincloud”), (ii) genes with longest 3′UTRs expressed in brain (*n* = 1261 probes) or (iii) genes with longest 3′UTRs expressed only in brain (*n* = 449 probes). We repeated the analysis for 1000 random samples of 192 genes annotated with the GO-term “Ion channel activity” (GO:0005216), which was the most significant *p*-value in enrichment analysis (*n* = 269 probes).

### Prediction of miRNA response elements

We used TargetScan (version 5.0; Lewis et al., 2005) to predict miRNA response elements (MREs) in all annotated constitutive and longest alternative 3′UTRs. For all constitutive and longest alternative 3′UTRs annotated in brain, we estimated the percentage of MREs expressed in adult FC (Landgraf et al., 2007) by dividing the number of MREs for miRNAs expressed in adult FC by the total number of MREs predicted for the region of interest. The difference between MRE percentages in longest alternative 3′UTRs compared to constitutive 3′UTRs in the brain was assessed using a Kolmogorov-Smirnov test (KS-test). We repeated the analysis considering only families containing miRNAs whose expression was 10-times, 5-times, or 2-times higher than the median expression across all non-neuronal tissues (Landgraf et al., 2007); hereafter referred to as FC10, FC5, and FC2 sets. We included only *ion channel/transporter genes* which had at least 1 MRE predicted in alternative and constitutive 3′UTRs (*n* = 209). The same analysis was done for the remaining *n* = 300 genes expressing the longest alternative 3′UTRs only in brain after removing *ion channel/transporter genes*.

Next, we compared the percentage of shared MREs for FC10, FC5 and FC2 (Landgraf et al., 2007, Supplementary Table S1) predicted in constitutive and alternative 3′UTRs for each pair of *ion channel/transporter genes* (*n* = 226). Precisely, we compared the observed median value to what would be expected based on the median percentage of shared MREs between 1000 random sets of *n* = 226 genes expressed in the brain, excluding *ion channel/transporter genes*.

## Results

### Annotation and characterization of human genes 3′untranslated regions

Long poly-A^+^ selected single- and paired-end RNA sequencing data from 16 normal human tissues was used to annotate transcripts *de novo* using Cufflinks (version 1.3.0, Trapnell et al., 2010, Figure 1A). For simplicity, we only considered genes whose complete set of protein-coding transcripts contain the same constitutive stop codon. Exonic nucleotides downstream of these stop codons form part of these protein-coding transcripts 3′UTR. For each gene, we defined its constitutive 3′UTR as all 3′UTR nucleotides expressed across the complete set of transcripts. The remaining 3′UTR nucleotides were defined as contributing to alternative 3′UTRs. For each gene we also defined its longest alternative 3′UTR as its alternative 3′UTR with the largest number of nucleotides observed in any tissue (Figure 1B).

We next sought to compare the density of functional elements in alternative and constitutive 3′UTRs using the derived allele frequency (DAF) test (Chen and Rajewsky, 2006). A fraction of SNPs with low allele frequencies that is higher than found for SNPs in neutrally evolving sequence suggests that negative selection has acted to prevent the accumulation of loss-of-function mutations, an evolutionary signature of functionality (Chen and Rajewsky, 2006). We compared the ratio, *r*, of rare (DAF < 10%) to intermediate (10% < DAF < 90%) frequency SNPs (Clarke et al., 2012; Haerty and Ponting, 2013) found in constitutive or longest alternative 3′UTRs against the ratio calculated for neutrally evolving sequences. As neutral proxy we used transposable element-derived sequences (Ancestral Repeats or ARs, Lunter et al., 2006) that inserted prior to the common ancestor of human and mouse. To ensure that ARs shared the same phylogenetic history as the 3′UTRs, we selected ARs in regions in the vicinity of 3′UTRs (20, 50, or 100 kb) (Haerty and Ponting, 2013). 3′UTRs without ARs in their vicinity were excluded (*n* = 4324 genes with longest alternative 3′UTRs contained ARs within 20 kb vicinity). Both longest alternative (*r* = 15304/6334 = 2.42; two tailed Fisher's exact test (FET) *p*-value < 7 × 10^−3^) and constitutive (*r* = 12900/5144 = 2.51; FET *p*-value < 2 × 10^−5^) 3′UTRs exhibit a moderate excess of SNPs at low frequency (DAF < 10%) relative to ARs within 20 kb (*r* = 18143/7928 = 2.29), consistent with some nucleotides in these regions being preferentially preserved by natural selection. Constitutive 3′UTRs were no different to longest alternative 3′UTRs in this test (FET *p*-value = 0.10), suggesting that they evolved under similar selective constraints. Similar results were obtained when ARs within 50 kb and 100 kb either up- or down-stream of 3′UTRs were considered. We conclude that 3′UTR extensions are subject to selective constraint in humans, consistent with these regions harboring functionally conserved motifs.

**Figure 2:**
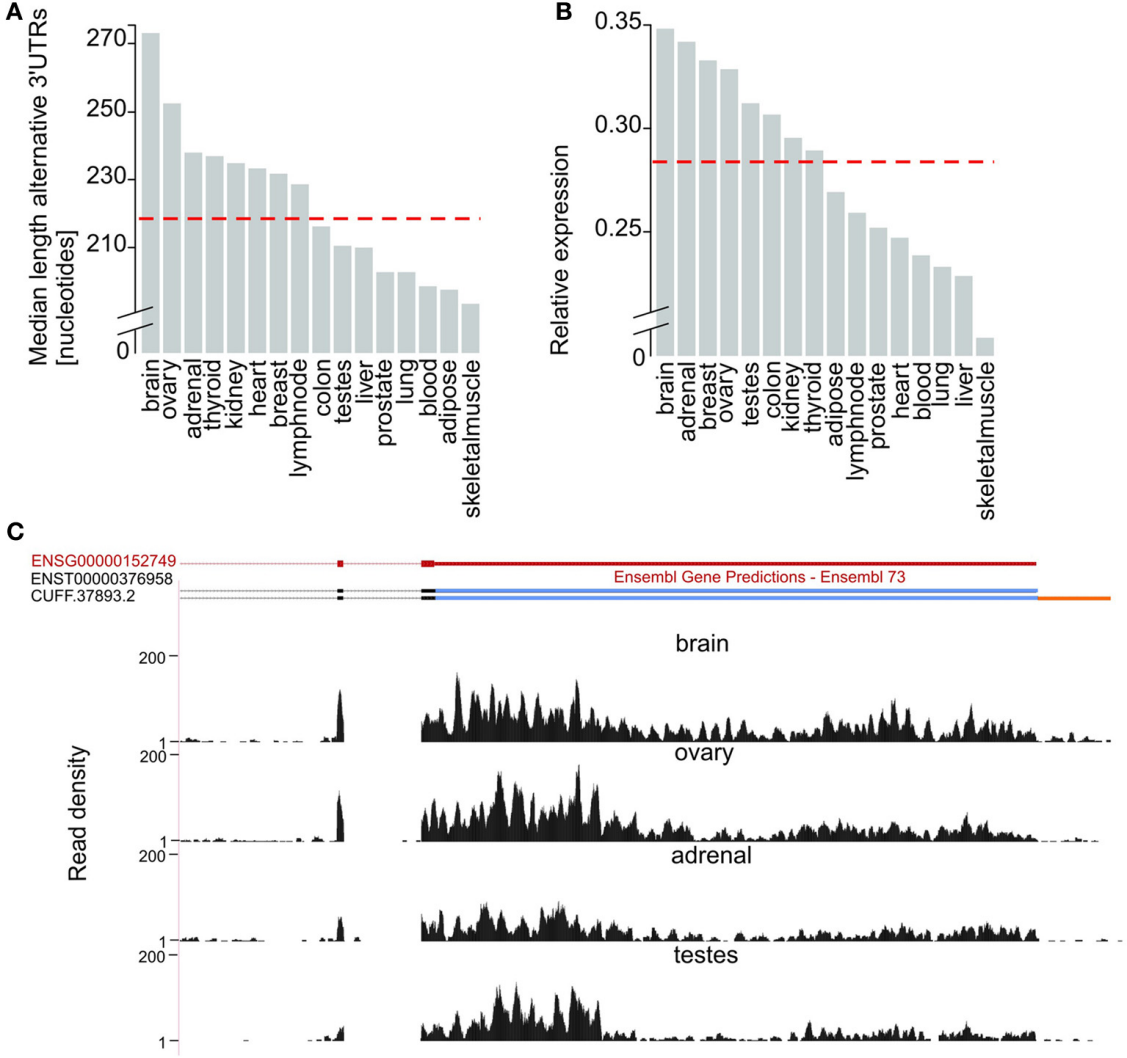
**Brain expressed isoforms have the longest 3′UTR extensions. (A)** Median length (in nucleotides, Y-axis) of alternative 3′UTRs across 16 human tissues. The median across all non-neuronal tissues (219 nucleotides) is depicted as a horizontal dashed red line. **(B)** Median fold difference in expression (calculated as the number of reads per nucleotide of expressed sequence) of alternative relative to constitutive 3′UTR (Y-axis) across 16 tissues. The median across all non-neuronal tissues (median relative expression = 0.28) is depicted as a horizontal dashed red line. **(C)** G protein-coupled receptor 180 (*GPR180*; ENSG00000152749; chr13:95,277,556-95,288,768, red) expresses its longest 3′UTR in the brain. Alongside support for the expression of the only transcript annotated by ENSEMBL for this gene (ENST00000376958) our approach allowed the annotation of an isoform with an extended 3′UTR in the brain (CUFF.37893.2). Read density (black, Y-axis) across the last coding exons (black), the constitutive (blue) and extended (orange) portions of *GPR180* 3′UTR in brain, ovary, adrenal and testes.

Next, we evaluated tissue differences in 3′UTR usage. Alternative 3′UTRs expressed in the human adult brain are significantly longer than those found in other tissues (two-tailed Wilcoxon test, *p*-value < 5 × 10^−8^), consistent with previous reports (Hilgers et al., 2011; Miura et al., 2013). The median length of brain-expressed alternative 3′UTRs (272 nucleotides) is 8% higher than for ovary (251 nucleotides, Figure 2A), the tissue with the next longest median alternative 3′UTRs, and 24% higher than the median length of 3′UTRs of transcripts from all other tissues (219 nucleotides, Figure 2A). The observed extension of brain-expressed alternative 3′UTRs could reflect a brain-specific usage of distal poly-adenylation signals or splice sites or be a consequence of technical bias, such as differences in RNA library preparation or sequencing coverage. To distinguish between these two explanations, we compared the ratio between the expression levels of alternative and their corresponding constitutive 3′UTRs, across all tissues tested. Relative Expression level (*E*) was defined as the ratio between the numbers of reads per nucleotide in alternative and constitutive 3′UTRs (Methods). We found that isoforms expressed in the brain have the highest relative expression of alternative compared to constitutive 3′UTRs (Figures 2B,C). More specifically, the median relative expression level of all genes with annotated alternative 3′UTRs was significantly higher in the brain (*E* = 0.35) than the median relative expression of the other 15 tissues investigated (*E* = 0.28; Figure 2B; Wilcoxon test, *p*-value < 2.2 × 10^−16^). This is consistent with brain-expressed transcripts having the longest alternative 3′UTRs owing to biological rather than technical reasons.

### Isoforms with long alternative 3′UTRs are often co-expressed and functionally related

We next tested whether genes expressing isoforms with the longest alternative 3′UTRs in the brain are functionally related, contributing to the same biological process or being part of the same pathway. To do so we compared the frequency of GO annotations for the 544 genes that express their longest alternative 3′UTRs only in the brain against GO-terms for all brain expressed genes with annotated 3′UTRs.

**Figure 3:**
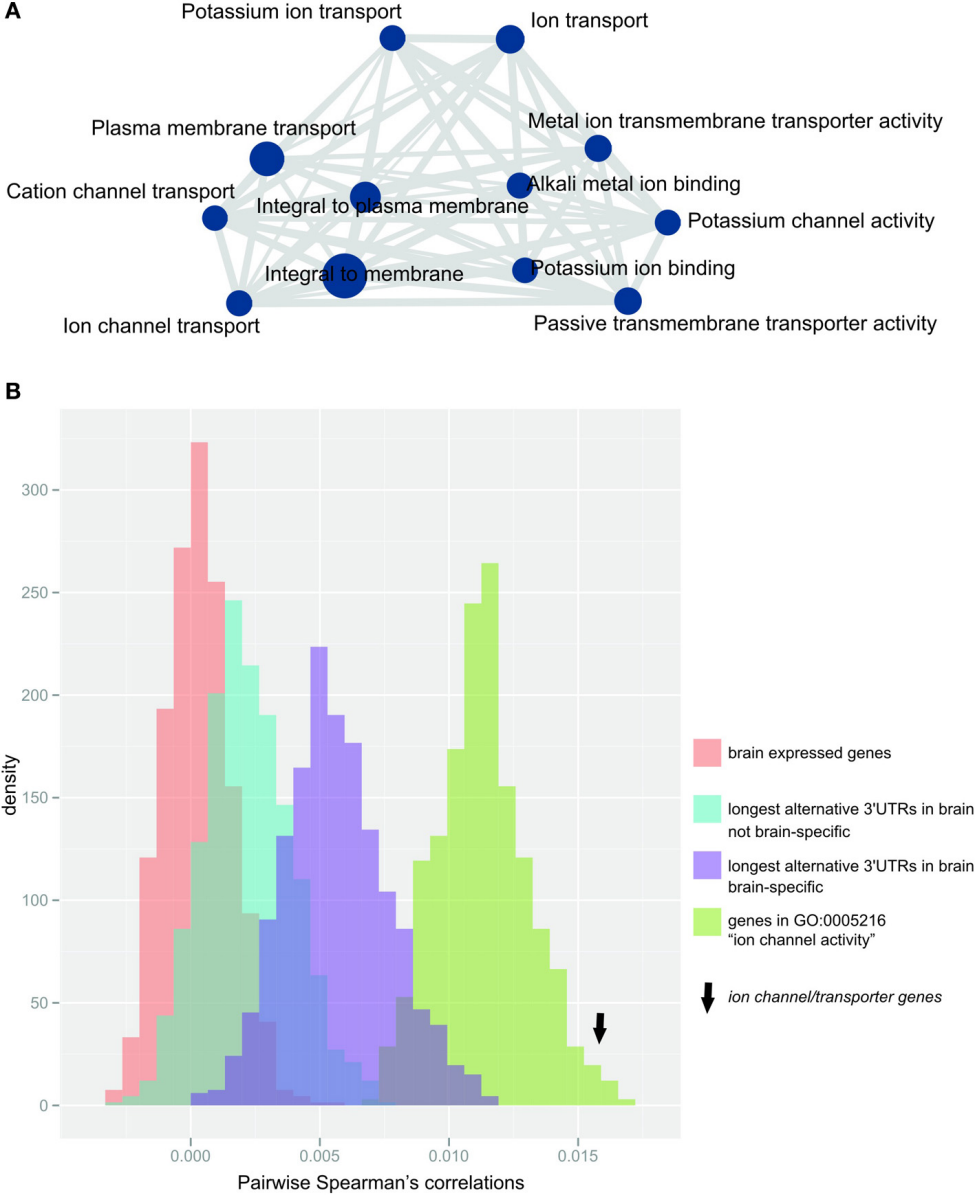
**Ion channel/transporter genes are over-represented within genes expressing their longest 3′UTR in the brain (A) Enrichment map (Merico et al., 2010) of significantly enriched GO-terms after terms were summarized using REVIGO (Supek et al., 2011)**. Node size (blue circles) reflects the number of contributing genes, and edge size (gray lines) corresponds to the number of genes that are shared between nodes. **(B)** Distribution of the median pairwise Spearman's correlations for 1000 bootstrap samples of random gene sets (each *n* = 192, matched to *ion channel transporter genes*) from the populations: (i) brain-expressed genes (*n* = 16812 probes; red), (ii) genes with longest alternative 3′UTRs expressed in brain (*n* = 1261 probes; blue), (iii) genes with longest 3′UTRs expressed only in brain (*n* = 449 probes; purple) or (iv) genes contributing to GO-term “ion channel activity” (*n* = 192 probes; green). Arrow indicates the median pairwise Spearman's correlations for *ion channel/transporter genes*.

After correcting for multiple testing (Bonferroni correction, α = 0.05), we found that genes with the longest alternative 3′UTR isoform expressed in the brain were significantly enriched in 28 GO-terms that were related to either ion channel or transporter function, as summarized using REVIGO (Supek et al., 2011; Figure 3A, Supplementary Table S1). Almost half of the genes expressing their longest 3′UTR in the brain contributed to the observed enrichment (*n* = 226 genes; Supplementary Table S2). The protein-coding genes that contribute to this enrichment are, for simplicity, hereafter termed *ion channel/transporter genes*. None of the terms that were enriched for genes with longest alternative 3′UTRs in brain was replicated in any of the other 15 tissues tested (Supplementary Table S3). *Ion channel/transporter genes* tended to express especially long alternative 3′UTRs, showing a median length of *n* = 1322 nucleotides compared to *n* = 798 in the remaining *n* = 322 genes with longest 3′UTRs only in brain (two-tailed Wilcoxon test, *p*-value < 0.05).

Relative to non-related genes, the expression of genes functioning in the same pathway or sharing biological roles is expected to be more strongly co-regulated. Indeed, we found that *ion channel/transporter genes* (*n* = 192 genes associated with probes in the arrays used by Colantuoni et al., 2011) showed significantly increased pairwise correlation in expression across the PFC of postmortem brains from 148 healthy adult subjects (Colantuoni et al., 2011) compared to randomly selected gene sets of brain expressed genes of the same size (median Spearman's correlation = 0.015; *empirical p*-value < 0.001, Figure 3B). Similar results were found when we compared *ion channel/transporter genes* to random sets of genes expressing their longest 3′UTRs in the brain (*n* = 1261 probes; *empirical p*-value < 0.001, Figure 3B) or only in the brain (brain-specific; *n* = 449 probes; *empirical p*-value < 0.001, Figure 3B). The expression of genes contributing to the same GO-terms are more likely to be correlated with each other than random sets of brain expressed genes. For that reason, we compared *ion channel/transporter genes* to random sets of brain expressed genes annotated with the GO-term “ion channel activity” and found significantly increased correlation for *ion channel/transporter genes* (*n* = 269 probes; *empirical p*-value < 0.05, Figure 3B). The increased correlation in expression of *ion channel/transporter genes* indicates that extended alternative 3′UTRs facilitate coordination of these genes in the brain.

### Long alternative 3′UTRs strengthen miRNA mediated cross-talk among *ion channel/transporter genes* in the brain

Our finding that the expression levels of *ion channel/transporter genes* show increased correlation in the human adult brain, prompted us to investigate the mechanism underlying these genes' coordinated expression. We hypothesized that alternative 3′UTRs might contain MREs for miRNAs that are shared between *ion channel/transporter genes* thereby enhancing the post-transcriptional coordination of their relative abundance in the adult PFC, by means of miRNA mediated cross-talk between transcripts.

We compared the percentages of predicted MREs per gene in constitutive or longest alternative 3′UTRs of *ion channel/transporter genes* for those miRNA families that are known to be expressed in the human adult FC (Landgraf et al., 2007). We defined sets of miRNA families whose miRNAs are transcribed (denoted “expressed”) or specifically expressed in the FC at levels that are at least 10-, 5-, or 2-times (denoted FC10, FC5, and FC2, respectively) higher than their median level across all non-neuronal tissues (Landgraf et al., 2007; Supplementary Table S1). For each gene, MRE percentage was defined as the ratio of predicted MREs for miRNA families expressed in or specific to the FC divided by the total number of predicted MREs. Only *ion channel/transporter genes* with at least one predicted MRE in alternative and constitutive 3′UTRs were considered (*n* = 209 genes).

**Figure 4:**
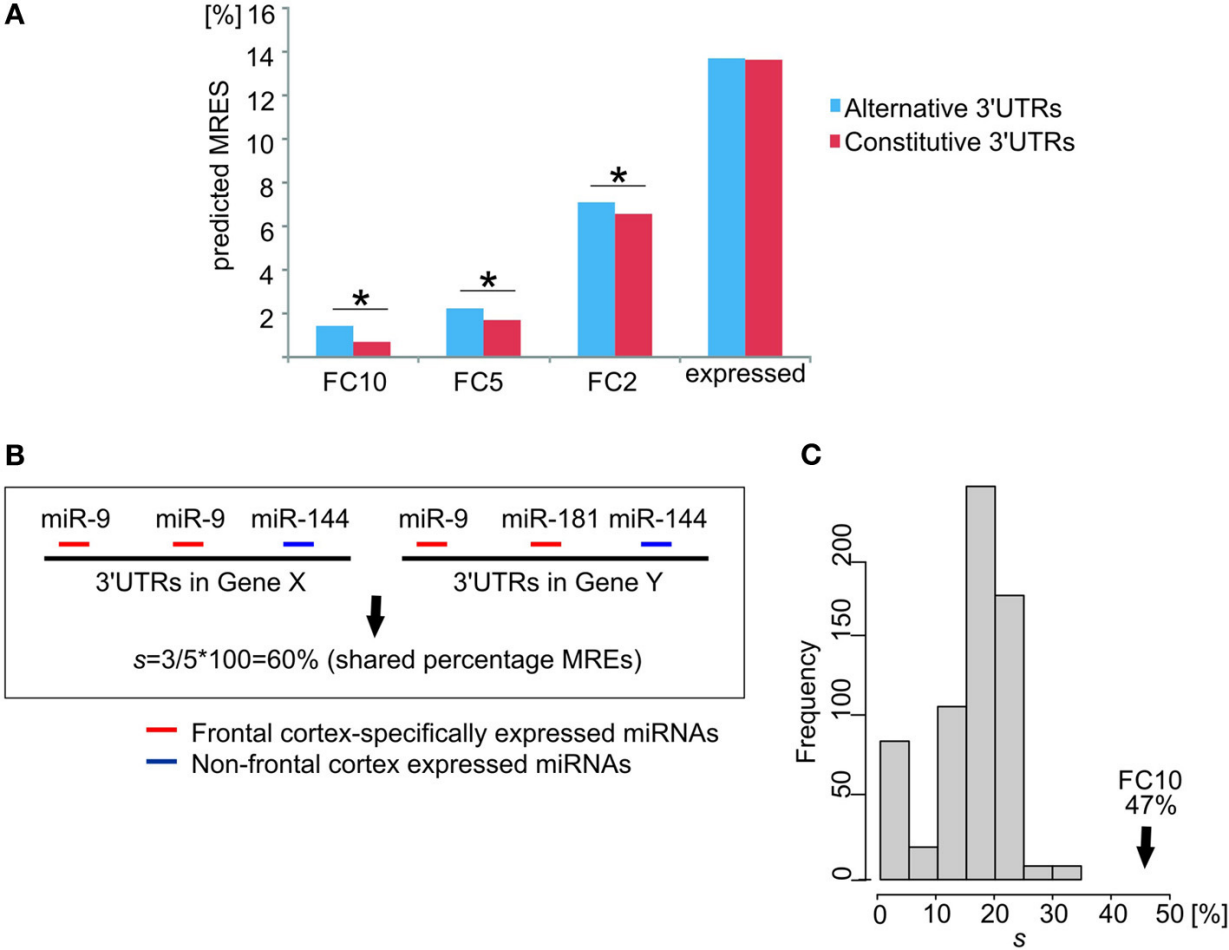
**Frontal cortex specifically expressed miRNAs targeting 3′UTR extensions strengthen the cross-talk between *ion channel/transporter genes*. (A)** Differences in the percentage of predicted MREs for miRNA families expressed in human adult frontal cortex (y-axis for different levels of relative expression: FC10, FC5, FC2, and expressed) between constitutive (red) and alternative (blue) 3′UTRs of *ion channel/transporter genes*. ^*^ indicates a significant difference (*p* < 0.05). **(B)** Calculation of the percentage (%) of shared MREs, *s*, between two *ion channel/transporter* genes. Gene X and Gene Y have two and one predicted MREs for a frontal cortex-specific miRNA (miR-9; red), respectively. Gene X and Y have predicted MREs for miRNAs that are either not expressed specifically in the frontal cortex (Gene X, miR-144) or not shared (Gene Y, miR-181). Of the 5 predicted MREs in both genes 3 are for frontal cortex-specific miRNAs (miR-9; red), resulting in *s* = 3/5 × 100 = 60% shared MREs. **(C)** Percentage shared MREs for FC10 in *ion channel/transporter genes* (median percentage shared MREs *s* = 47%, black arrow). Histogram represents the null distribution of the same metric for 1000 random brain-expressed gene sets excluding *ion channel/transporter genes* (each *n* = 226).

Longest alternative 3′UTRs for *ion channel/transporter genes* expressed in the brain showed a significantly higher percentage of MREs for miRNA families that are specifically expressed in the human FC relative to constitutive 3′UTRs (Figure 4A). This observation for *ion channel/transporter genes* is consistent with a recent report of a general enrichment of neuronal specific miRNAs across 3′UTR extensions in the human brain (Miura et al., 2013). The difference in MRE percentage for alternative or constitutive 3′UTRs widened for increased tissue specificity of miRNA families in the human FC (Figure 4A). For the FC10 set, the median percentage of MREs predicted in brain alternative 3′UTRs was twice as high as for constitutive 3′UTRs. This large increase in the percentage of MRE for specifically expressed miRNAs was not found in genes expressing their longest 3′UTRs only in brain after removing *ion channel/transporter genes* (*n* = 300 genes, two-sample KS-test *p*-value = 0.45).

We hypothesized that competition for shared miRNAs contributes to the observed increased coordination in expression between *ion channel/transporter genes*. To test this hypothesis, we calculated the proportion, *s*, of all MREs that were predicted to bind FC-specific miRNAs and that were present within the constitutive and alternative 3′UTRs of a pair of *ion channel/transporter genes* expressing their longest 3′UTR in the brain and shared between the two genes 3′UTRs; we will refer to this as the “percentage of shared MREs” (Figure 4B). The median of *s* for these *ion channel/transporter genes* was then compared to values from randomly selected brain-expressed genes. *Ion channel/transporter genes* were found to share a significantly (*empirical p-value* < 0.001) higher percentage of predicted MREs with each other (median *s* = 47% for FC10 miRNA families) than other brain-expressed genes (Figure 4C). The median *s* value between gene-pairs was highest for miRNA families that were specifically expressed in the frontal cortex (FC10 set). Values decreased to 40% (*p-value* < 0.001) for FC5 miRNA families, and to 33% for FC2 miRNA families (*p-value* < 0.001) as would be expected if the most FC-specific miRNAs contributed the most to the observed coordination of *ion channel/transporter gene* transcript abundance.

**Figure 5:**
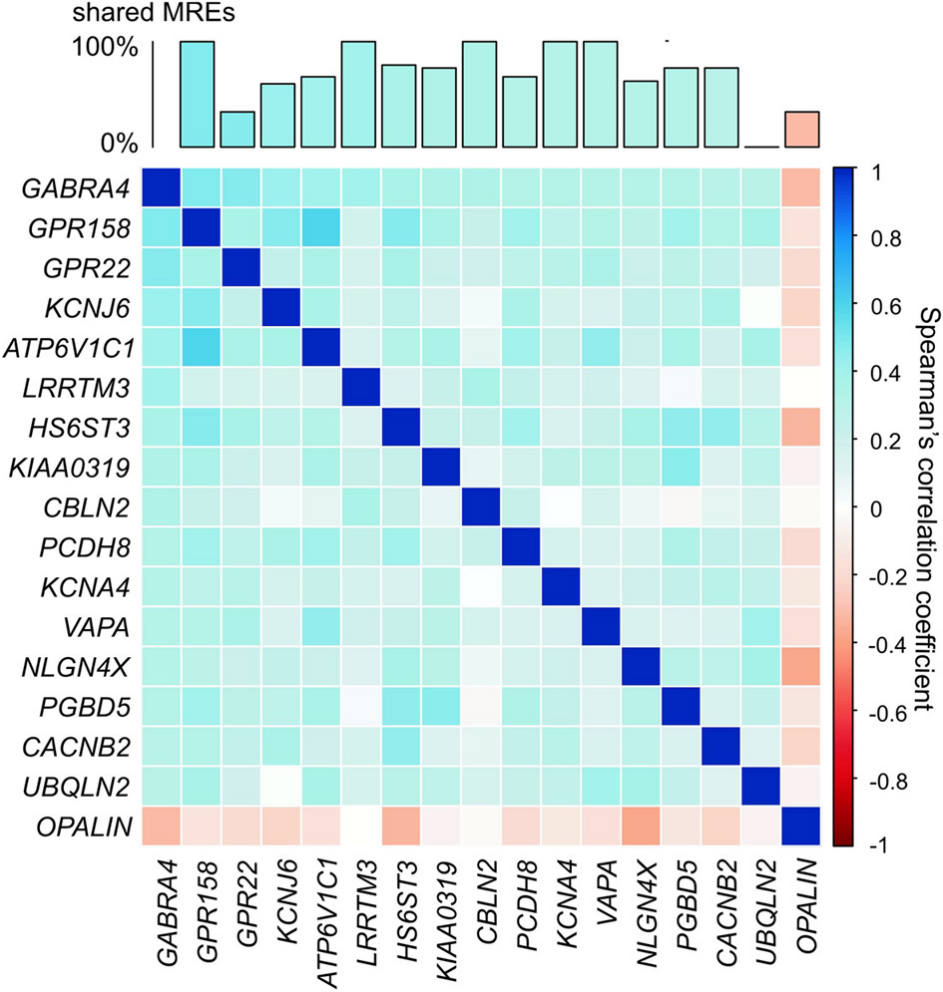
***GABRA4*-subnetwork**. Example of gene in ion channel/transporter network. Correlation matrix of *ion channel/transporter genes* significantly correlated with *GABRA4* (α = 0.05, Bonferroni corrected, pairwise Spearman's correlation). Bar chart shows percentage of MREs *GABRA4* shares with every other gene in *GABRA4*-subnetwork.

*GABRA4* (Gamma-aminobutyric acid receptor, alpha-4) is an appropriate illustration of the ion channel/transporter network described above. *GABRA4* belongs to the receptor gene family GABA-A of ion channels and interacts with GABA, the main inhibitory neurotransmitter in the brain (Whiting et al., 1999). The expression level of *GABRA4* has been linked to learning and long-term potentiation (Shen et al., 2010) and its expression levels are increased following seizures (Roberts et al., 2005). We identified *ion channel/transporter genes* that show significantly correlated expression (α = 0.05; Bonferroni corrected) in the adult human brain with *GABRA4* (Figure 5). The genes highly co-expressed with *GABRA4* included those encoding potassium channels which are involved in nervous excitability (Pongs, 1999) and activity-dependent regulation of neuronal signaling (e.g., *KCNB1*, Park et al., 2006), as well as G-protein coupled receptors (e.g., *GPR158*) which are crucial for many physiological and disease pathways (Patel et al., 2013). Significantly co-expressed genes with *GABRA4* share a large number of MREs for FC specifically expressed miRNAs (Figure 5) consistent with miRNA-mediated crosstalk between *ion channel/transporter genes* in the *GABRA4*-subnetwork potentially contributing to their coordinated expression.

## Discussion

The biological relevance of the widespread and conserved tendency of protein-coding genes to express isoforms with longer alternative 3′UTRs in brain (Hilgers et al., 2011; Miura et al., 2013) has been poorly understood. Given the role of these regions in the post-transcriptional modulation of relative transcript abundance by means of miRNA mediated cross-talk (Salmena et al., 2011), we hypothesized that long alternative 3′UTRs could enhance the miRNA-regulated abundance of functionally related genes. To test this hypothesis, we used mRNA sequencing data to annotate 3′UTRs in adult human tissues. Consistent with previous analysis in humans (Hilgers et al., 2011; Miura et al., 2013) and other animals (Zhang et al., 2005; Ji et al., 2009; Ulitsky et al., 2012) we find that brain-expressed isoforms have longer alternative 3′UTRs which are enriched for brain-expressed miRNA binding sites (Miura et al., 2013). Our results are not a consequence of limited coverage or other technical bias or limitations resulting from the choice of experimental data set, because expression of extended, relative to constitutive, 3′UTRs is significantly higher in the brain than in other adult tissues. By comparing the expression of alternatively and constitutively expressed nucleotides within the same genes we thus controlled for potential limitations arising from limited depth of sequencing or differences in library preparation. Two major novel findings emerged from our analysis of genes expressing their longest alternative 3′UTRs in the brain. Firstly, that ion channel/transporter genes are enriched within this gene set; secondly that these genes exhibit increased transcriptional coordination mediated by competition for shared FC-specific miRNAs.

Transport across the cellular membrane is primarily mediated by ion channels and other transporter genes and this function is crucial to ensure appropriate responses to changing extra- and intracellular environments. In the brain, transport across the cellular membrane is crucial for cellular excitability. Controlled responses to both excitatory and inhibitory signals that underlie communication between cells in the brain require the dynamic regulation of the abundance and repertoire of ion channel and other transporter proteins (Voglis and Tavernarakis, 2006). This is achieved at several levels including transcriptional and post-translational control. For example, in neurons some ion channel/transporter gene encoding transcripts are enriched at dendrites (Cajigas et al., 2012). Translation of transcripts localized at dendrites is evolutionarily conserved in multiple species (Cajigas et al., 2012) and is believed to allow efficient spatial and temporal resolution of extracellular cues (Matsumoto et al., 2007; Andreassi and Riccio, 2009).

Translation of localized mRNAs is tightly regulated post-transcriptionally by miRNAs, with several brain-expressed miRNAs present within axonal compartments (Loohuis et al., 2012). These miRNAs are capable of actively regulating local mRNA translation (Manakov et al., 2009; Smalheiser and Lugli, 2009). Importantly, miRNA expression and repertoires are highly sensitive to microenvironmental changes, with several miRNAs exhibiting increased transcription, for example upon long term potentiation (Park and Tang, 2009). The observations that both ion channel/transporter genes and miRNAs respond to changes in environment and have specific and overlapping subcellular localizations suggest that their interactions mediate responses to milieu changes. Transmembrane transport that is mediated by ion channels/transporters, in response to changes in cellular microenvironment results in changes in miRNA abundance that in turn will translate in post-transcriptional repression of their targets, including transporter protein-encoding mRNAs. This regulatory feedback loop is likely to provide dynamic yet robust changes in ion channel/transporter abundance (Jiang et al., 2012).

Our findings that a subset of ion channel/transporter genes tend to express longer alternative 3′UTRs in the brain and that the sequence of these extensions is enriched for FC-specific miRNA response elements is consistent with this proposal. Furthermore we show that predicted MREs located at alternative 3′UTRs can enhance the miRNA mediated crosstalk between these transcripts, likely contributing to the observed coordination of ion-channel/transporter genes in the brain.

We propose that the alternative 3′UTRs of ion channel/transporter mRNAs in the brain post-transcriptionally enhance the robustness of dynamic chances of their product abundance in response to differences in cellular microenvironment ensuring coordinated assembly of functional transmembrane channels.

### Conflict of interest statement

The authors declare that the research was conducted in the absence of any commercial or financial relationships that could be construed as a potential conflict of interest.
